# Accurate Evaluation of Hepatocyte Metabolisms on a Noble Oxygen-Permeable Material With Low Sorption Characteristics

**DOI:** 10.3389/ftox.2022.810478

**Published:** 2022-06-06

**Authors:** Masaki Nishikawa, Hiroyasu Ito, Fumiya Tokito, Keita Hirono, Kousuke Inamura, Benedikt Scheidecker, Mathieu Danoy, Takumi Kawanishi, Hirohsi Arakawa, Yukio Kato, Katsuhiro Esashika, Hiroshi Miyasako, Yasuyuki Sakai

**Affiliations:** ^1^ Department of Chemical System Engineering, University of Tokyo, Tokyo, Japan; ^2^ Faculty of Pharmacy, Institute of Medical, Pharmaceutical and Health Sciences, Kanazawa University, Kanazawa, Japan; ^3^ Film & Sheet Materials Depatment, Functional Materials Laboratory, R&D Center, Mitsuichemicals, Inc., Tokyo, Japan; ^4^ Chemicals Safety Department, Responsible Care and Quality Assurance Div., Mitsuichemicals, Inc., Tokyo, Japan

**Keywords:** hepatocyte, oxygen supply, chemical sorption, drug development, toxicity test, 4-polymethyl-1-pentene polymer, PDMS

## Abstract

In the pharmaceutical industry, primary cultured hepatocytes is a standard tool used to assess hepatic metabolisms and toxicity *in vitro*. Drawbacks, however, include their functional deterioration upon isolation, mostly due to the lack of a physiological environment. Polydimethylsiloxane (PDMS) has been reported to improve the function of isolated hepatocytes by its high oxygen permeability when used as a material of microphysiological systems (MPS). However, its high chemical sorption property has impeded its practical use in drug development. In this study, we evaluated a new culture material, 4-polymethyl-1-pentene polymer (PMP), in comparison with PDMS and conventional tissue culture polystyrene (TCPS). First, we confirmed the high oxygen permeability and low sorption of PMP, and these properties were comparable with PDMS and TCPS, respectively. Moreover, using primary rat hepatocytes, we demonstrated maintained high levels of liver function at least for 1 week on PMP, with its low chemical sorption and high oxygen permeability being key factors in both revealing the potential of primary cultured hepatocytes and in performing an accurate evaluation of hepatic metabolisms. Taken together, we conclude that PMP is a superior alternative to both PDMS and TCPS, and a promising material for a variety of drug testing systems.

## 1 Introduction

The liver plays a central role in various metabolisms and maintains homeostasis of the body (Trefts et al., 2017). It is one of the main organs that regulate pharmacokinetics in the body and the prediction of liver metabolisms and toxicity has been of great interest in the pharmaceutical industry. For *in vitro* testing, human primary hepatocytes are considered to be the “gold standard,” but quick functional deterioration of primary hepatocytes upon isolation is a major obstacle realizing an accurate *in vitro* evaluation of liver metabolisms and toxicity.

Functional deterioration of liver function can be attributed to the lack of a physiological environment, such as interactions among parenchymal and non-parenchymal cells, oxygen supply, and physical and biochemical stimuli. Microphysiological systems (MPS), also known as organ-on-a-chip technology, have been proposed to recapitulate the *in vivo* physiological environment (Ribeiro et al., 2019; Baudy et al., 2020). Hepatocytes require a relatively greater amount of oxygen among the various cell types, and if cultured on gas-impermeable materials, they are found to adapt to hypoxic conditions by altering metabolisms (Scheidecker et al., 2020). Therefore, sufficient oxygen supply is critical to improve not only the hepatic function, but also various aspects of the hepatocyte culture, including initial cell attachment, survival rate, cell density, and interaction with non-parenchymal cells, and in unison, those factors can further ameliorate hepatic function (Scheidecker et al., 2020). Perfusion of culture medium in both a conventional culture and MPS devices is known to facilitate the supply of oxygen and other nutrients to cultured hepatocytes. Furthermore, it can naturally create an oxygen gradient along the direction of perfusion, which is a key factor to stimulate metabolic zonation, i.e., zonal identities of hepatocytes (Matsumoto et al., 2019). However, in order to meet the high oxygen demand of hepatocytes *in vitro* without oxygen carriers such as red blood cells, a high perfusion rate is required, and in turn, the cells are exposed to excessive shear stress. In contrast, simple but effective alternatives include direct oxygenation to cultured hepatocytes through oxygen-permeable materials placed at the bottom surface. This approach, together with multi-gas incubators, is also known to be able to induce metabolic zonation, although it is not in a single device as is possibly achieved by perfusion devices (Scheidecker et al., 2020). Oxygen supplementation with this methodology can therefore substitute excessive perfusion rates and shows potential for a standard platform of high throughput simple testing systems.

Polydimethylsiloxane (PDMS) is an inexpensive and biocompatible silicone elastomer with high oxygen permeability. It is most widely used for MPS devices due to its rapid prototyping and compatibility with imprinting processes for fabrication. High oxygen permeability of PDMS considerably improves the cell culture environment and function of hepatocytes not only in MPS devices but also in multi-well culture plates where the conventional polystyrene bottom surface is replaced with PDMS (Nishikawa et al., 2008a; Xiao et al., 2014; Scheidecker et al., 2020). However, a major drawback of this material is the substantial sorption–not only adsorption to the surface but also absorption to the inner bulk material–of hydrophobic molecules. It was confirmed that multiple compounds, such as Nile Red could penetrate into PDMS, with fluorescence increasing due to accumulation during extended exposure time (Toepke and Beebe, 2006). Wang et al. focused on the octanol/water partition coefficient, logP, of compounds and quantitatively measured the sorption of five small molecules on PDMS (Wang et al., 2012). The sorption of more hydrophilic compounds than Phenytoin (logP = 2.47) was almost negligible (less than 20%), while the sorption of more hydrophobic compounds than Rhodamine 6G (logP = 2.62) were substantial (more than 80%). This indicates that logP is an important factor affecting the sorption on PDMS. Grant et al. successfully simulated drug concentrations in PDMS microfluidic devices using experimentally determined diffusion and partition coefficients of drugs in PDMS (Grant et al., 2021). However, it is also shown that the sorption on PDMS was not determined exclusively by hydrophobicity (van Meer et al., 2017), indicating experimental measurements are unavoidable to determine the degree of sorption of different chemicals. In particular, many of the drugs used in *in vitro* liver experiments are hydrophobic molecules, and a strong sorption on PDMS leads to a decrease in the concentration of the target drugs, which compromises the accuracy and reliability of the test system (Hirama et al., 2019; Zhou et al., 2019).

Various types of surface modifications and coatings have been proposed to inhibit the sorption of molecules into PDMS (Gokaltun et al., 2017). In order to inhibit the non-specific adsorption of macromolecules such as proteins, attempts have been made to coat the surface of PDMS with polymers such as PEG (Makamba et al., 2005; Wu et al., 2006; Zhou et al., 2009). However, this technique cannot inhibit the internal sorption of hydrophobic small molecules. Sasaki et al. reported another attempt that the sorption of Rhodamine B could be inhibited by coating the surface of PDMS with parylene (Sasaki et al., 2010). However, since parylene is a gas impermeable material, its disadvantage is that the coating blocks oxygen permeation through PDMS. The most effective technique to inhibit sorption on PDMS is probably the sol–gel method. In this method, a solution containing SiO_2_ or TiO_2_ is poured onto PDMS and made to react to a gel state via solvation, and then dried to coat the surface with fine particles (Roman et al., 2005; Roman and Culbertson, 2006; Abate et al., 2008; Orhan et al., 2008). Sjoberg et al. demonstrated the effect of coating with a sol–gel method by investigating toxicities of anticancer drugs on fibroblasts cultured in PDMS devices. They showed that in uncoated devices, the drug concentration decreased due to sorption and the cells survived, whereas in coated devices, the drug concentration was maintained, and cell death occurred. They also showed that the oxygen permeability did not change before and after treatment (Gomez-Sjoberg et al., 2010). Thus, sorption onto PDMS can be suppressed to some extent by surface treatments. However, besides the cost and labor, the possibility of degradation of the coating presents a major disadvantage for using these surface treatments in actual applications. In order to solve these problems, a new material that is oxygen permeable and less sorptive is desired.

In the scope of constructing a simple *in vitro* drug test system using primary hepatocytes, we evaluated a new culture material, 4-polymethyl-1-pentene polymer (PMP), which is a proprietary material supplied by Mitsuichemicals, Inc. First, we confirmed the high oxygen permeability and low sorption of PMP. Then, we cultured primary rat hepatocytes on PMP and evaluated liver functions including various drug metabolisms. The results were compared with those of PDMS and conventional tissue culture polystyrene (TCPS) culture systems.

## 2 Materials and Methods

### 2.1 Cell Culture Plates

PMP plates used in this experiment were made by attaching 50 μm thick PMP sheets provided by Mitsui Chemicals, Inc. to polystyrene frames of a 24-well plate format (Vecell, Japan). A double-sided adhesive tape for silicone rubber from innovect was used for attachment. PMP plates were exposed to plasma for 1 min and coated with a 2-fold diluted aqueous solution of collagen type I-P (Nitta Gelatin, Japan). Collagen-coated TCPS plates were purchased (IWAKI, Japan). PDMS plates were coated with collagen as reported previously (Nishikawa et al., 2008b; Xiao et al., 2014). Briefly, PDMS plates were treated with plasma, and coupled with aminosilane (Shinetsu Silicone, Japan). The introduced amino groups were made to react with the cross-linker N-(4-Maleimidobutyryloxy) succinimide (GMBS; Dojindo, Japan) and were coated with collagen type 1-P (Nitta Gelatin, Japan). The amount of adsorbed collagen was quantified by using the BCA protein assay kit (Thermo Fisher Scientific, United States).

### 2.2 Cell Culture and Animal Handling

Primary rat hepatocytes were isolated by standard two-step collagenase perfusion method from 8-week-old male Wistar rats. Isolated hepatocytes were subsequently purified by a series of washes with a serum-free EMEM medium (Gibco, United States), followed by a Percoll (GE Healthcare, Sweden) gradient purification as previously described (Xiao et al., 2014). Immediately after the isolation, cells were suspended in a fresh culture medium (Supplementary Table S1) and plated at a density of 1.0 e5 cells/cm^2^ on different types of plates described previously. On the basis of the previous report (Scheidecker et al., 2020), PMP and PDMS plates were incubated in an incubator with 10% oxygen to approximate the metabolisms and functions *in vivo*, while TCPS plates were incubated in an incubator with 20% oxygen to minimize the adverse effects of hypoxia. After inoculation, primary rat hepatocytes were washed with a fresh culture medium for 4 h. From day 1 to day 7, the medium was replaced every other day with a medium containing Matrigel at low concentration (150 μg/ml), expecting a certain amount of deposition and accumulation of Matrigel on the cell surface. The cell viabilities on different types of plates were evaluated on day 3 by Calcein AM, ethidium homodimer-1, and Hoechst 33342 staining (Dojindo Laboratories), according to the manufacturer’s instruction. Animals were kept at a 12 h light/dark cycle with free access to water and a standard chow diet. Animal handling and the experimental setup were in accordance with the protocols and guidelines approved by the University of Tokyo (approval number 2706).

### 2.3 Oxygen Permeability Test

On day 3, the oxygen concentration near the cell layer was measured using an oxygen sensor without medium replacement. REDFLASH-based fluorescent oxygen probe (PyroScience, United States) was connected to a FireSting O_2_ logger (PyroScience, United States) and used by following the manufacturer’s instruction. The probe was lowered onto the cell layer, and the oxygen tension (pO_2_) values were measured and recorded at a steady-state. The oxygen consumption of the cells was calculated from the difference between the measured oxygen concentration and that in the incubator and the oxygen permeability coefficient using Fick’s law (Nishikawa et al., 2008a; 2008b).

### 2.4 Sorption Test

Fluorescent molecules that differ only in functional groups: Rhodamine 123, Rhodamine B, and Rhodamine 6G, were used as representatives of hydrophobic low-molecular-weight compounds. The initial concentration of the solution of Rhodamine 123 was 5 μM, and those of Rhodamine B and Rhodamine 6G were 10 μM in PBS(−). Those solutions were placed in wells of plates as described previously without collagen coating. At 4, 24, and 48 h after exposure, the solutions were stirred well and sampled 50 μl at a time. The amount of sorbed substrate was determined by measuring the concentration in the collected sample with a fluorescent reader. After 48 h, the plates were washed, and the sorption was observed using a confocal microscope.

### 2.5 Hepatic Function Test

#### 2.5.1 Albumin Secretion

The amount of albumin secretion of hepatocytes was measured by sampling 50 μl of the medium during medium exchange on days 1, 3, 5, and 7. Albumin concentration was measured by ELISA and the amount secreted was calculated from the absorbance read by a plate reader.

#### 2.5.2 Gene Expression Analysis

On days 3 and 7, cells were washed with 0.5 ml/well of PBS(−), and RNA samples were collected with 0.35 ml/well of Trizol (Thermo Fisher Scientific, United States). RNA extraction was performed using a silica column-based RNA extraction kit (Zymo Research, United States), and the quality and quantity of total RNA were measured by NanoDrop (Thermo Fisher Scientific, United States). Total RNA was converted to cDNA with a PrimeScript reverse transcriptase kit (Takara, Japan). The expression levels were assessed by quantitative real-time PCR using KOD SYBR Green chemistry (Takara, Japan) in the StepOnePlus (Applied Biosystems, United States) system. The list of genes measured is shown in Supplementary Table S2 with sequences of primers which were designed with Primer-BLAST and purchased from Eurofins Genomics. The expression levels were normalized by that of β-actin as an internal control.

#### 2.5.3 CYP Activity Test

The CYP activity test was performed as described previously (Kozakai et al., 2014; Scheidecker et al., 2020; Shinohara et al., 2021). The CYP substrate mixture used in this experiment contained 20 μM Phenacetin, 2 μM Coumarin, 5 μM Bupropion, 0.1 μM Amodiaquine, 1 μM diclofenac, 40 μM Mephenytoin, 5 μM Bufuralol, and 2 μM Midazolam for CYP1A2, CYP2A6, CYP2B6, CYP2C8, CYP2C9, CYP2C19, CYP2D6, and CYP3A4, respectively (Table 1). Hepatocytes were cultured with the mixture on days 3 or 7. In addition, 0.5 ml/well of the medium containing the substrate cocktail was added to the wells without cells, and the amount of sorption to the culture materials was evaluated. Sampling was conducted after 4, 24, and 48 h of exposure. Aliquots of the exposed culture medium (30 µL) were mixed with 5 µL of water and 115 µL of methanol containing 1 µM of niflumic acid as an internal standard. After vortex mixing, the solutions were centrifuged at 21,500×*g* for 5 min at 4°C, and the resulting supernatants were injected into the LC–MS/MS system. The amount of each metabolite was determined using an LCMS8050 triple quadrupole mass spectrometer (SHIMADZU CORPORATION, Japan) coupled with an LC-30A system (SHIMADZU CORPORATION). Chromatography was performed on a CAPCELL PAK C18 MG III column (ID 2.0 × 50 mm; Osaka Soda Co. Ltd., Osaka, Japan) at 50°C through step-gradient elution with a flow rate of 0.4 ml/min according to the following program: 0–0.5 min, 95% A/5% B; 0.5–3.0 min, 95% A/5% B to 20% A/80% B; 3.0–4.0 min, 20% A/80% B; 4.0–4.1 min, 20% A/80% B to 95% A/5% B; 4.1–5.5 min, 95% A/5% B (A, water containing 0.1% formic acid; B, acetonitrile containing 0.1% formic acid). The detected mass numbers and collision energy (CE) was as follows, acetaminophen for CYP1A2 (152.0 > 110.0, CE: −9 V), 7-hydroxycoumarin (7-OH Coumarin) for CYP2A6 (163.0 > 107.0, CE: −24 V), hydroxybupropion (OH Bupropion) for CYP2B6 (256.0 > 238.0, CE: −13 V), N-desethylamodiaquine for CYP2C8 (328.0 > 283.0, CE: −18 V), 4′-hydroxydiclofenac (4′-OH Diclofenac) for CYP2C9 (312.0 > 230.0, CE: −32 V), 4′-hydroxymephenytoin (4′-OH Mephenytoin) for CYP2C19 (235.1 > 150.1, CE: −17 V), 1′-hydroxybufuralol (1′-OH Bufuralol) for CYP2D6 (278.0 > 186.0 CE: −19 V), 1′-hydroxymidazolam (1′-OH Midazoram) for CYP3A4 (342.0 > 203.0, CE −27 V), and niflumic acid (283.2 > 265.2, CE: −21 V). The analytical standard curve for each metabolite was made using commercially available products. Lab solutions software (version 5.89, SHIMADZU CORPORATION) was used for data manipulation.

**TABLE 1:** CYP Enzymes, substrates, and metabolites.

Enzymes	Substrates	Metabolites
CYP1A2	Phenacetin (20 µM)	Acetaminophen
CYP2A6	Coumarin (2 µM)	7-OH coumarin
CYP2B6	Bupropion (5 µM)	OH bupropion
CYP2C8	Amodiaquine (0.1 µM)	N-desethylamodiaquine
CYP2C9	Diclofenac (1 µM))	4-OH diclofenac
CYP2C19	(S)-Mephenytoin (40 µM)	4-OH mephenytoin
CYP2D6	Bufuralol (5 µM)	1-OH bufuralol
CYP3A2	Midazolam (2 µM)	1-OH Midazolam

#### 2.5.4 Statistical Analysis

All values were expressed as mean ± S.D. from multiple independent experiments as indicated in the figure legend. ANOVA followed by Tukey’s honestly significant difference (HSD) test was performed for multiple comparisons unless stated otherwise. Pairwise comparisons were conducted via Student’s *t*-test in Supplementary Figure S5.

## 3 Results

### 3.1 Oxygen Permeability Test

**FIGURE 1:**
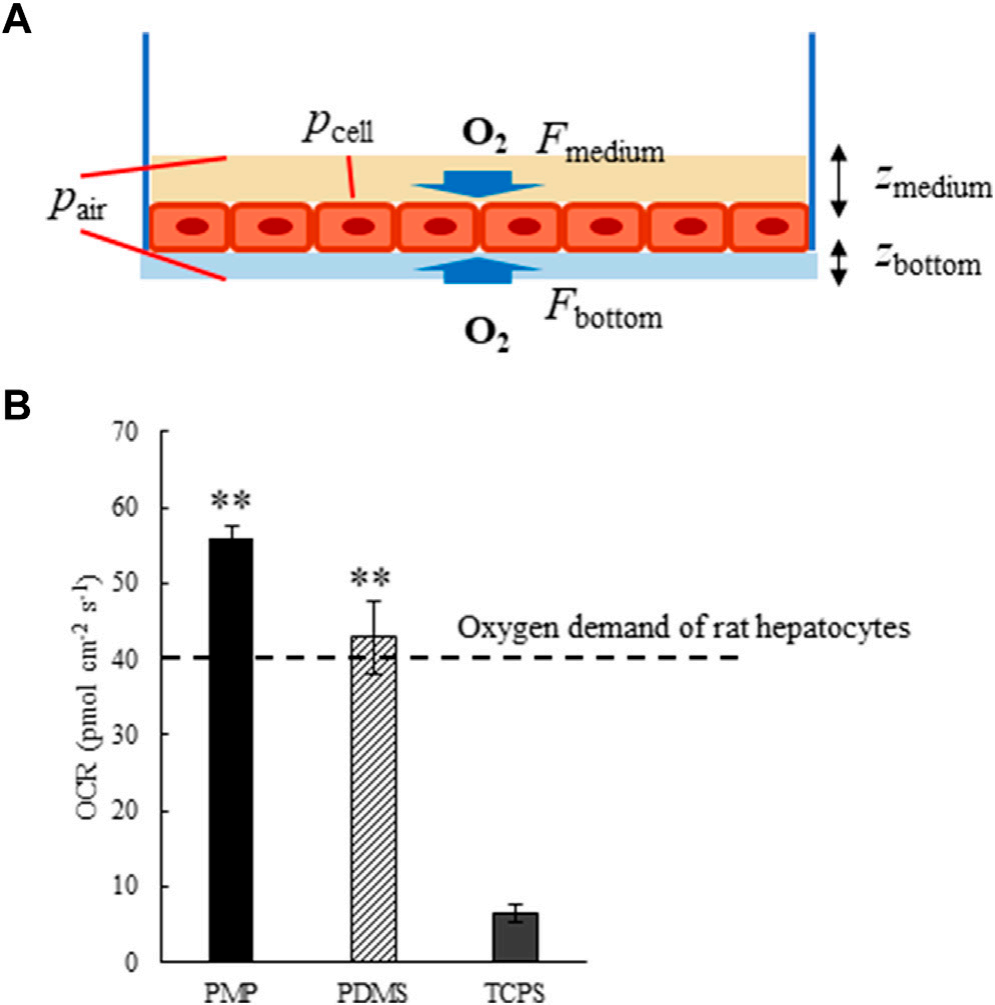
**(A)** Schematic diagram of Oxygen consumption rates (OCR) calculation. *F*: Oxygen flux in the medium (*F*_medium_) or materials of the bottom surface (*F*_bottom_), *p*: Oxygen partial pressure at the cell layer (*p*_cell_) or in the air (*p*_air_), *z*: Thickness of the medium (*z*_medium_) or materials of the bottom surface (*z*_bottom_). **(B)** Oxygen consumption rates (OCR) of primary rat hepatocytes cultured on PMP, PDMS, and TCPS plates (**: *p* < 0.01, N = 3, vs. TCPS).

The parameters and the schematic diagram of the oxygen consumption rate (OCR) calculation are shown in Table 2; Figure 1A. In the case of PMP and PDMS, oxygen is supplied not only from the medium but also from the bottom surface, so, both were calculated and then added together. By assuming that the oxygen concentration near the cell layer is zero, the theoretical maximum oxygen supply rate can be calculated by using Fick’s law. OCRs in PMP and PDMS wells were calculated in a 10% oxygen atmosphere, while OCR in TCPS was calculated in a conventional 20% oxygen atmosphere. As the result, the theoretical maximum oxygen supply rate of PMP was about 2.0E2 (Table 3). This was about a half of PDMS, but it indicated that PMP could supply about 15 times more oxygen than TCPS. Next, we cultured rat primary hepatocytes in these wells and calculated OCRs of cultured hepatocytes based on the measurement of oxygen concentration near the cell layer. Compared with TCPS, OCRs were significantly greater in PMP and PDMS plates. After initial attachment, the stably cultured hepatocytes consumed 0.4 nmol/s/1E6 cells of oxygen. Therefore, as shown in Figure 1B, the oxygen demand of hepatocytes when seeded at 1E5 cells/cm^2^ can be calculated about 40 pmol/cm^2^/s. These results showed that both the calculation of maximum oxygen flux and the actual OCRs of hepatocytes satisfied the oxygen demand of hepatocytes in both PMP and PDMS cultures.

**TABLE 2:** Parameters for calculating OCR.

	PMP (E)	PDMS	Medium
Thickness [cm]	5.0–3	3.5E–2	2.6E–1
Oxygen permeation [pmol cm^−2^S^−1^ mmmHg^−1^]	1.3–2	1.3E–1^[1]^	2.6E–2^[2]^

**TABLE 3:** Maximum flux of oxygen in PMP, PDMS, and TCPS plates.

	PMP (E)	PDMS (E)	Medium (E)
Maximum flux [pmol cm^−2^s^−1^]	2.02	3.82	1.41

### 3.2 Sorption Test

**FIGURE 2:**
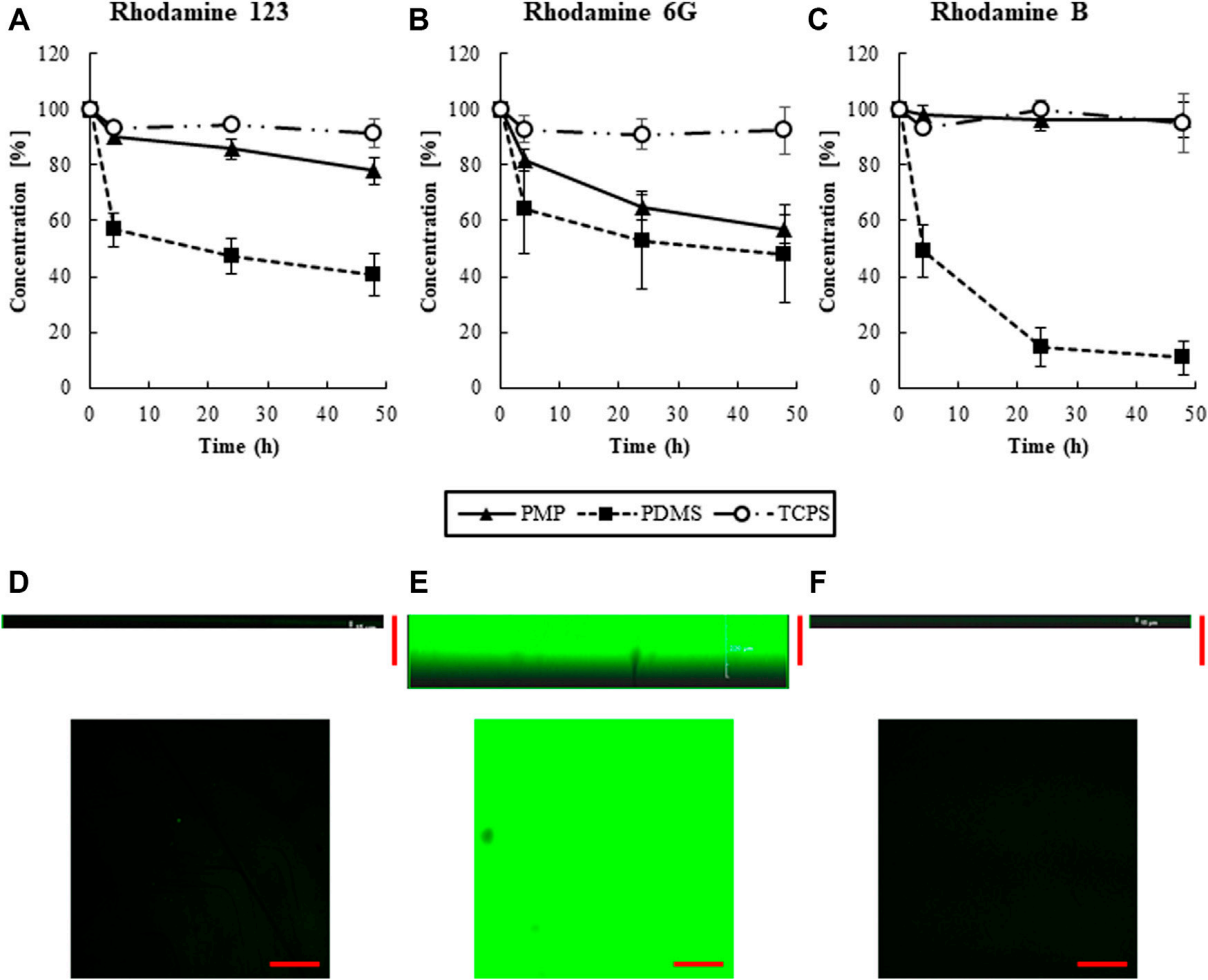
The transition of the concentrations of **(A)** Rhodamine 123, **(B)** Rhodamine 6G, **(C)** Rhodamine B, respectively (N = 6). Cross section of 48 h after adding Rhodamine B solution. **(D)** PMP, **(E)** PDMS, and **(F)** TCPS. Scale bars represent 200 μm.

Rhodamine 123, 6G, and B solutions were added to the wells with different types of materials, i.e., PMP, PDMS, and PDMS, and the change of concentration of the supernatant was measured to evaluate the amount of sorption as shown in Figures 2A–C, Supplementary Figure S1. A substantial amount of the chemicals was sorbed onto PDMS in all three chemicals. In PMP, although sorption of Rhodamine 6G was not so small, much less sorption was observed than in PDMS overall. Particularly, Rhodamine B was sorbed nearly 90% on PDMS plates, while just about 10% was sorbed on PMP and TCPS.

The cross-sectional view of PMP, PDMS, and TCPS were observed by confocal microscopy 48 h after the exposure of Rhodamine B to the wells (Figures 2D–F). Almost no fluorescence was observed in TCPS and PMP, whereas strong fluorescence was observed in PDMS up to around 200 μm from the surface. This supports the fact that intense internal sorption occurred in PDMS, but almost no sorption occurred in PMP and TCPS.

### 3.3 Hepatic Function Test

#### 3.3.1 Cell Morphology and Albumin Secretion

**FIGURE 3:**
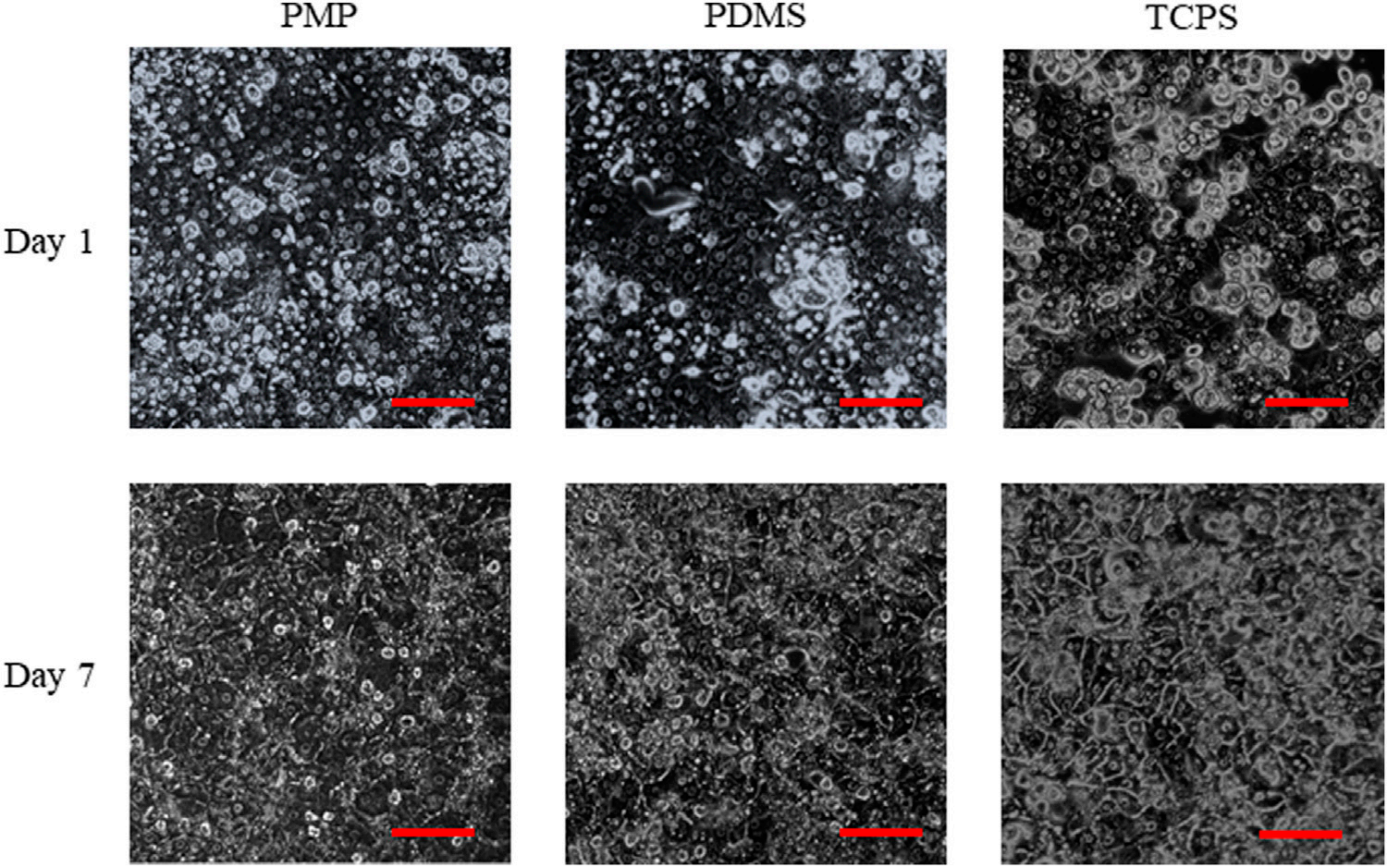
Morphologies of rat primary hepatocytes. Scale bars represent 100 μm.

**FIGURE 4:**
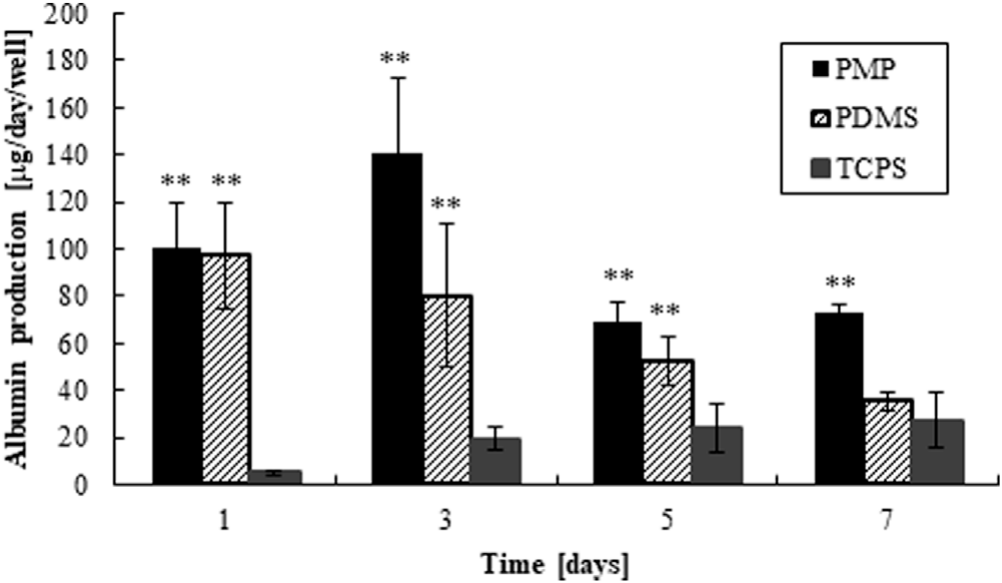
Albumin production of hepatocytes cultured on PMP, PDMS, and PDMS plates, respectively (**: *p* < 0.01, N = 6, vs. TCPS).

The amounts of adsorbed collagen on PMP and PDMS were quantified about 5–10 μg/cm^2^ (1.8–3.6 × 10^–11^ mol/cm^2^), which were enough to cover the surfaces in multi-layers of collagen molecules (Nishikawa et al., 2008b). The morphology of fresh rat hepatocytes on days 1 and 7 is shown in Figure 3. Almost all cells were alive as shown in Supplementary Figure S2, and monolayers were maintained in all conditions. The amount of albumin secretion measured by ELISA is shown in Figure 4. Albumin secretion was maintained at higher levels on PMP and PDMS compared to TCPS throughout the experimental period, although the difference between PDMS and TCPS was not significant on day 7.

#### 3.3.2 Gene Expression Analysis

**FIGURE 5:**
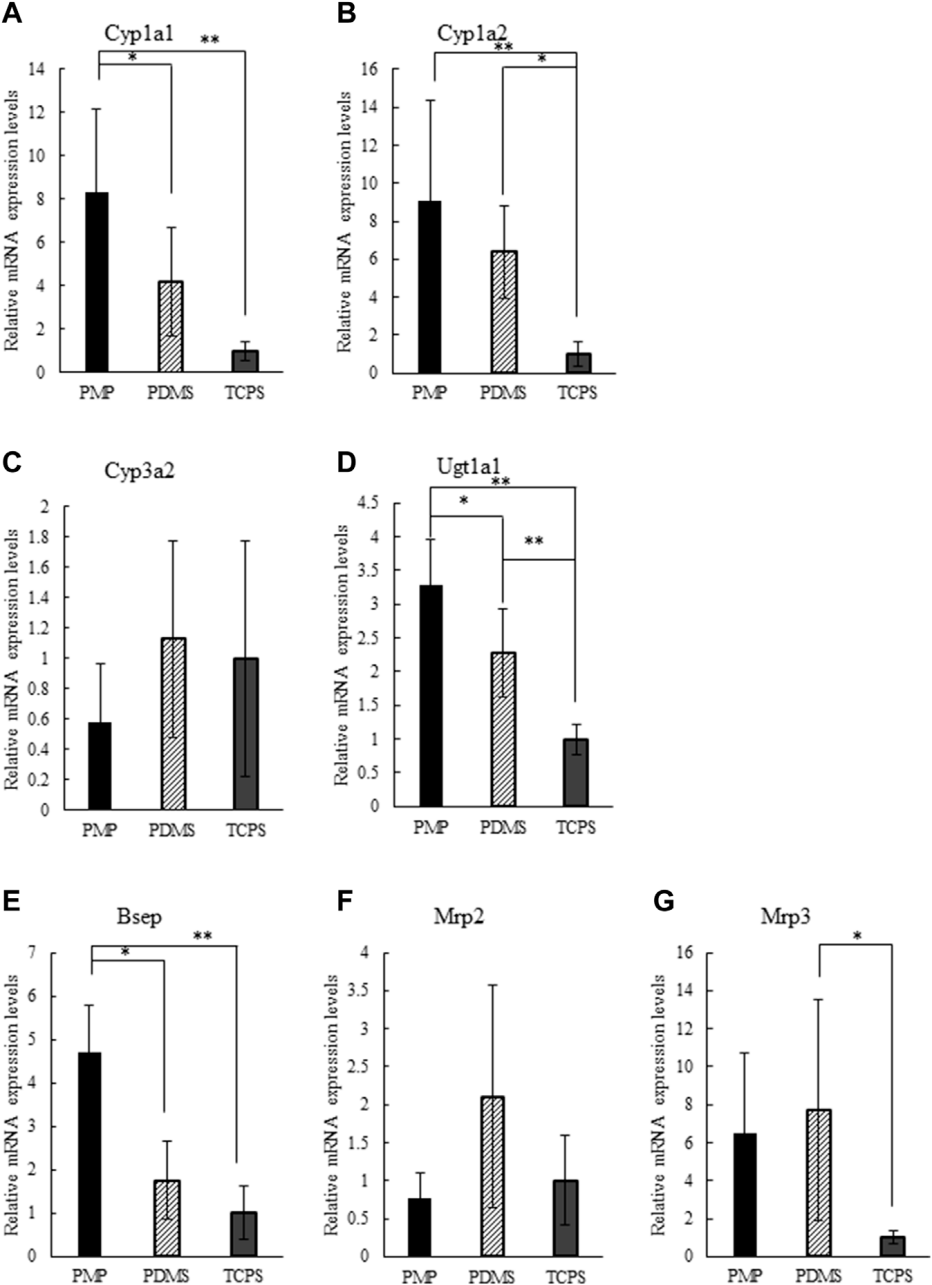
Relative gene expression levels on day 7 normalized by TCPS condition: **(A)** Cyp1a1, **(B)** Cyp1a2, **(C)** Cyp3a2, **(D)** Ugt1a1, **(E)** Bsep, **(F)** Mrp2, **(G)** Mrp3 (*: *p* < 0.1, **: *p* < 0.01, N = 6).

Expression patterns of genes related to drug metabolisms and kinetics were analyzed on days 3 and 7 and the results are shown in Supplementary Figures S3, S5, respectively. On day 7, PMP and PDMS showed significantly higher expressions of Cyp1a1, Cyp1a2, Ugt1a1, and Bsep than TCPS, and the expression levels of Cyp3a2, Mrp2, and Mrp3 were similar among the three culture conditions (Figure 5). In comparison with PDMS, PMP showed significantly higher expression levels of Cyp1a1, Ugt1a1, and Bsep, while the difference of expression levels of the other tested genes was not significant. In general, the expression levels of genes related to drug metabolisms and kinetics were higher in PMP and PDMS than in TCPS, presumably by direct oxygenation.

#### 3.3.3 CYP Activity Test

**FIGURE 6:**
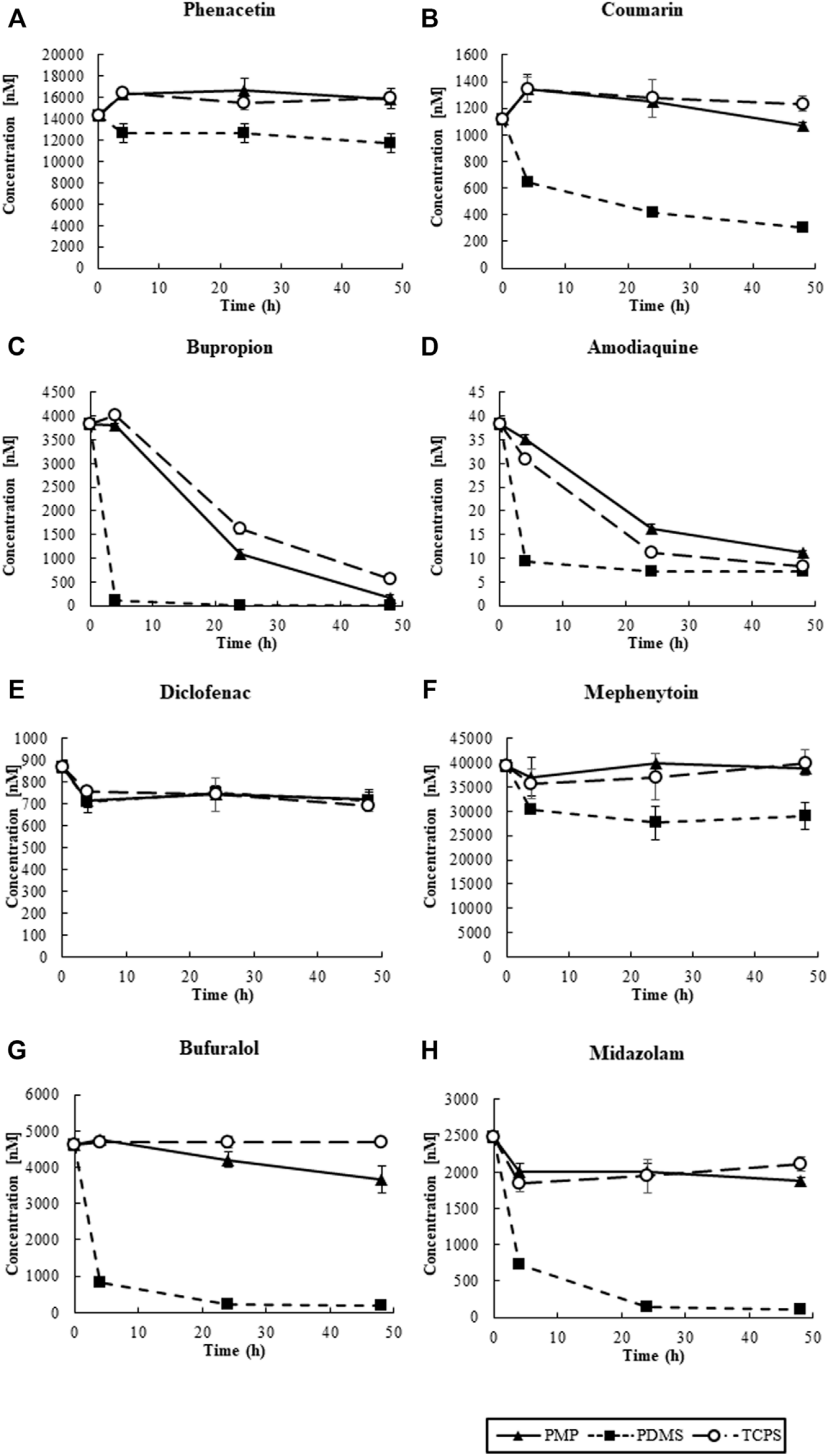
The transition of the concentrations of CYP substrates in solution: **(A)** CYP1A2, **(B)** CYP2A6, **(C)** CYP2B6, **(D)** CYP2C8, **(E)** CYP2C9, **(F)** CYP2C19, **(G)** CYP2D6, and **(H)** CYP3A2 (N = 4).

First, the substrate cocktail solution was added to the wells of PMP, PDMS, and TCPS without cells, and sorption of the substrates to materials was measured (Figure 6). At the starting time point, we observed misalignments of measured concentrations of chemicals, compared to the intended concentrations shown in Table 1. These results were anticipated as artificial and/or mechanical errors, such as dilution by remaining media in the wells. In PDMS wells, approximately 80% of Phenacetin, Diclofenac, and Mephenytoin were retained in the solution even after 48 h of exposure. However, only one-fourth of Coumarin and Amodiaquine remained in the solution, and most of Bupropion, Bufuralol, and Midazolam were sorbed into PDMS. Moreover, the sorption of these substrates into PDMS quickly happened for the first 4 h after exposure. In contrast, in the PMP and TCPS wells, most of the tested substrates except for Bupropion and Amodiaquine were retained in the solution for 48 h of exposure. Interestingly, although Bupropion and Amodiaquine were largely sorbed to both PMP and TCPS after 24 h, sorption at 4 h on both materials was almost negligible. Overall, the sorption to PMP was comparable with TCPS, and much less than that of PDMS.

**FIGURE 7:**
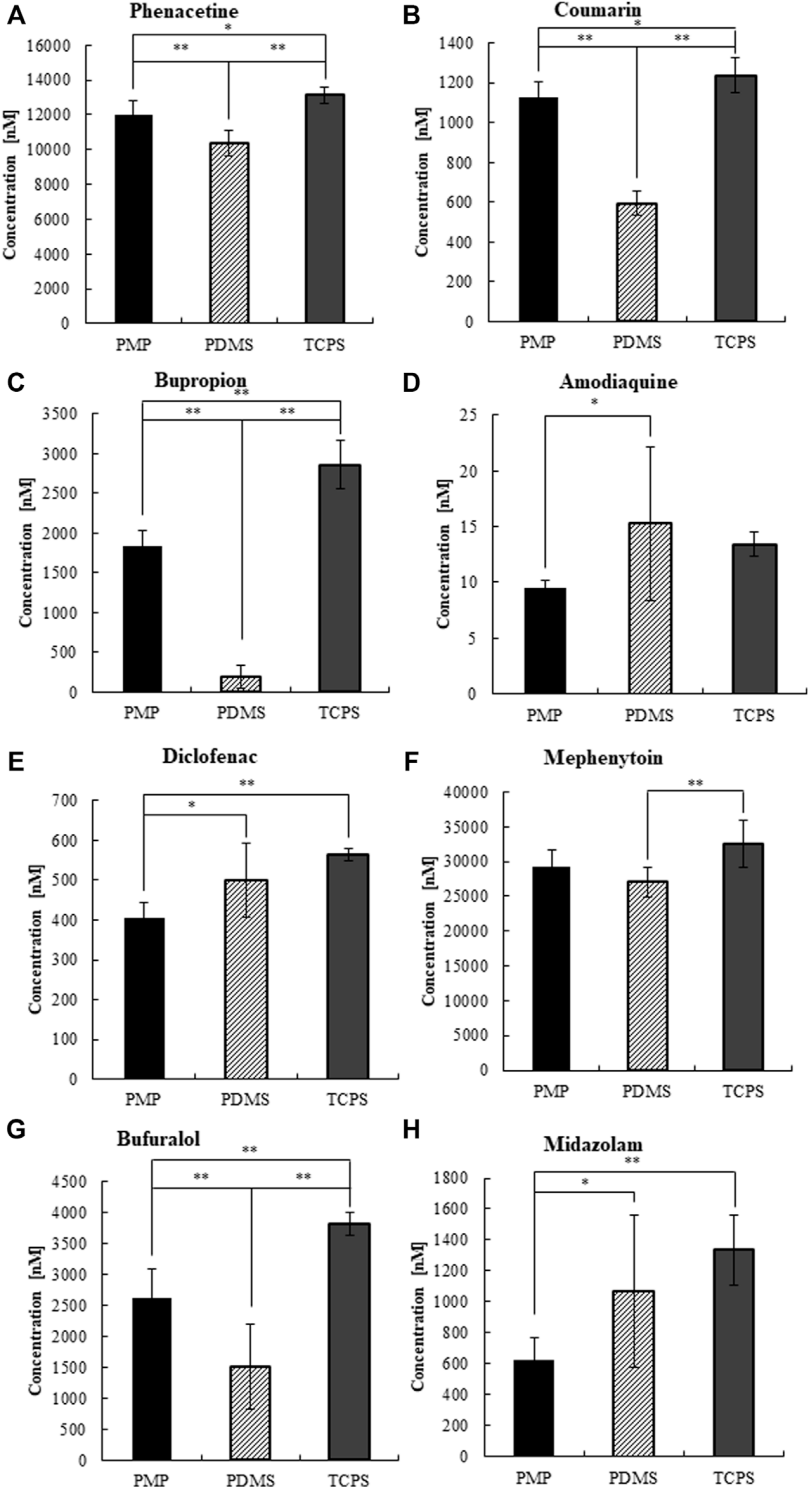
The concentrations of CYP substrates at 4 h of exposure to cultured hepatocytes on day 7.**(A)** CYP1A2, **(B)** CYP2A6, **(C)** CYP2B6, **(D)** CYP2C8, **(E)** CYP2C9, **(F)** CYP2C19, **(G)** CYP2D6, and **(H)** CYP3A2 (N = 8).

**FIGURE 8:**
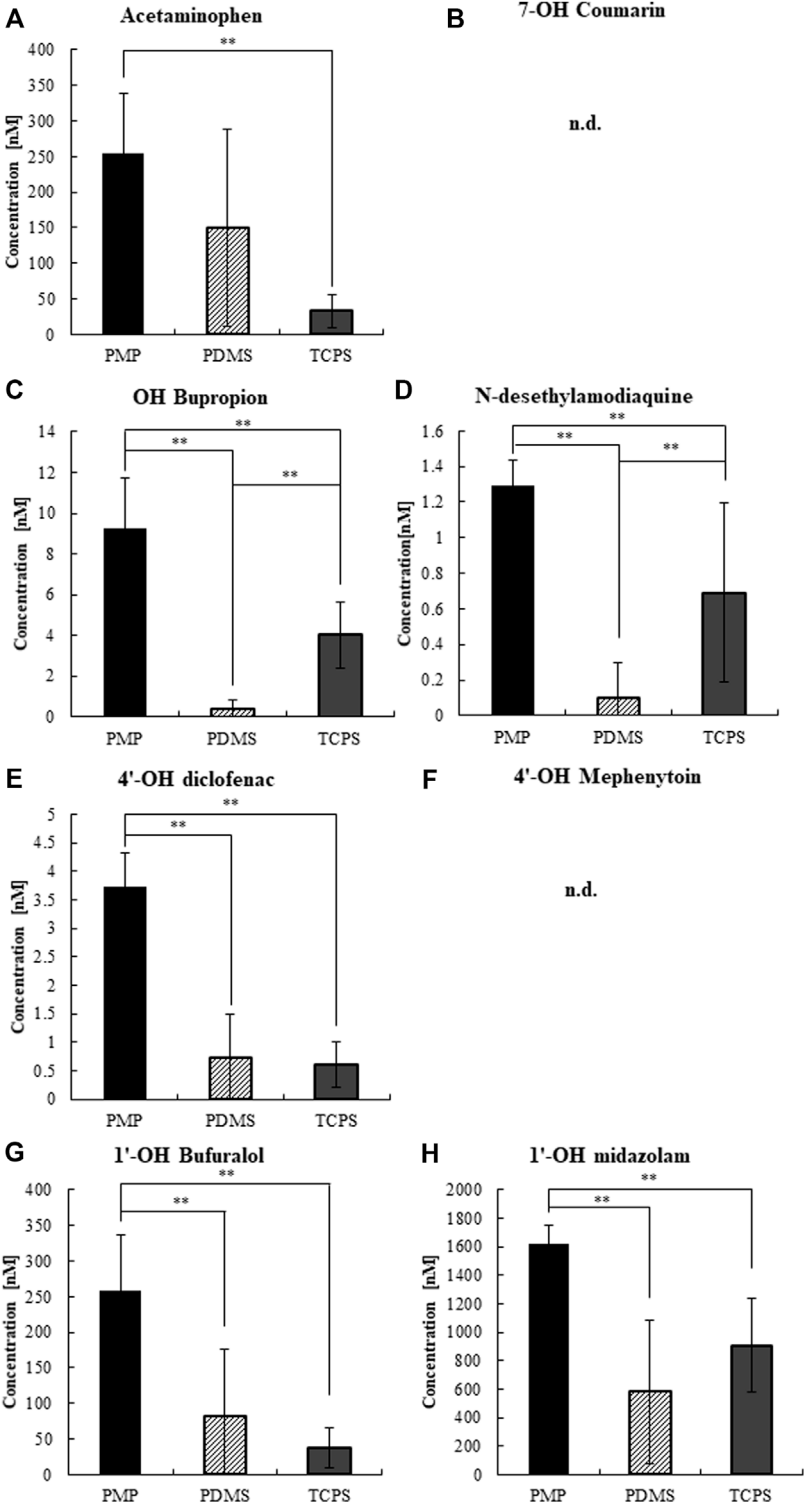
The concentrations of CYP metabolites at 4 h of substrate exposure to cultured hepatocytes on day 7. **(A)** CYP1A2, **(B)** CYP2A6, **(C)** CYP2B6, **(D)** CYP2C8, **(E)** CYP2C9, **(F)** CYP2C19, **(G)** CYP2D6, and **(H)** CYP3A2 (N = 8).

Next, we measured the CYP activity of hepatocytes cultured on those materials on day 3 (Supplementary Figures S4, S5) and day 7 (Figures 7, 8). On day 7, after 4 h of substrate exposure, at which the amount of sorption into PMP and TCPS were still not critical, the concentrations of substrates and their metabolites were measured. No metabolites of Coumarin (CYP2A6) and Mephenytoin (CYP2C19) were detected in this experiment (Figures 7B,F). Overall, almost all substrates were reduced more in PMP and PDMS than those in TCPS (Figure 7). Some substrates, such as Coumarin, Bupropion, and Bufuralol, showed a significant reduction in PDMS compared with PMP and TCPS (Figures 7B,C,G). However, they also showed a substantial sorption to PDMS (Figures 6B,C,G) and thus it is suggested that the pronounced reduction of those in PDMS were probably due to the loss by sorption, instead of cell metabolism. Among substrates which showed less sorption to all materials, the degree of reduction of Phenacetin (CYP1A2) and Diclofenac (CYP2C9) were comparable between PDMS and PMP and higher than that in the TCPS culture (Figures 7A,E). Interestingly however, when we compare the concentrations of their metabolites, they were detected significantly higher in PMP than in PDMS and TCPS (Figures 8A,E). As for the substrates which showed a substantial sorption to PDMS, such as Bupropion (CYP2B6), Amodiaquine (CYP2C8), Bufuralol (CYP2D6), and Midazolam (CYP3A4), their metabolites were detected much less in the PDMS culture than in the PMP culture (Figures 8C,D,G,H), and some of them were even less in PDMS than in TCPS (Figures 8C,D). Importantly, even as for substrates that showed comparable sorption to all materials, such as Phenacetine and Diclofenac, the concentrations of metabolites were less in PDMS and TCPS, and prominently high in the PMP culture (Figures 8A,E).

## 4 Discussion

In this study, we aimed to evaluate a new material, PMP, which has characteristics such as low chemical sorption and high oxygen permeability, in the scope of constructing a simple *in vitro* drug test system using primary hepatocytes. For this purpose, we performed an oxygen permeability test, chemical sorption test, and functional tests of primary hepatocytes, and demonstrated the superiority of the PMP culture system over PDMS and TCPS cultures.

In the oxygen permeability test, PMP satisfied the high oxygen demand of primary hepatocytes even in the 10% oxygen atmosphere as well as did PDMS, as expected by its physical properties. Previously, we have found that limited oxygen supply initiates an ischemic reprogramming resembling metabolic profiles of early-stage hepatocellular carcinoma, and also that the function and metabolisms of primary hepatocytes become closer to those in *in vivo* when direct oxygenation is achieved on PDMS under the 10% oxygen atmosphere than under a regular atmosphere (Scheidecker et al., 2020). Although many thermoplastics have been reported for cell culture, factors such an environment cannot be achieved according to their poor oxygen permeability (Gencturk et al., 2017). Even in “gas-permeable” thermoplastic elastomers (TPEs), such as a polystyrene-*block*-poly (ethylene butylene)-*block*-polystyrene triblock copolymer (SEBS) (Campbell et al., 2021; Schneider et al., 2021), the oxygen permeability is still not sufficient [22 of SEBS vs. 800 of PDMS; unit: × 10^–10^ cm^3^ (STP) cm cm^−2^ s^−1^ (cmHg)^−1^] to induce such metabolic phenotypes (Merkel et al., 2000; Uenishi et al., 2014). Together, it demonstrates the capability of PMP as an alternative to PDMS in regard to oxygen permeable materials.

The sorption test confirmed a substantial chemical sorption to PDMS, and showed almost comparable properties between PMP and TCPS (Figures 2,6). The images of confocal microscopy clearly showed that the internal absorption observed in PDMS was prevented in PMP and TCPS. Also, the transition of concentration of various molecules demonstrated that PMP was significantly less sorptive to various small molecules, compared with PDMS. However, the sorption of Rhodamine 6G in PMP was relatively high compared with TCPS, and other TPEs may hold some advantages in this regard (Schneider et al., 2021). Nonetheless, these results indicate that PMP is indeed a material with high oxygen permeability and low sorption. One of the purposes of using three different fluorescent substrates was to investigate the tendency of sorption due to different hydrophobicities. Since the hydrophobicity increases in the order of Rhodamine 6G, Rhodamine B, and Rhodamine 123, we assumed that the amount of sorption on PDMS would also follow this trend. However, the sorption of Rhodamine B was higher than that of Rhodamine 123. As reported previously (Wang et al., 2012; van Meer et al., 2017), this result suggests that, although logP is an important factor, sorption to PDMS is not determined exclusively by hydrophobicity. This renders the prediction of sorption to PDMS more difficult and new materials with low sorption characteristics more valuable.

Next, we cultured primary rat hepatocytes on PMP and tested the compatibility by evaluating the function of hepatocytes. Hepatocytes attached well on the collagen-coated PMP surface, and a healthy monolayer was maintained for at least a week. A gene expression analysis showed that the expression levels of genes related to drug metabolisms and kinetics were generally higher in PMP and PDMS than in TCPS. Albumin secretion, a basic indicator of the hepatic function, stayed at a high level compared with PDMS and TCPS cultures throughout the experimental period. In TCPS, the amount of albumin secretion was gradually increased during the culture period, while it decreased especially in PDMS. This may be explained by the combined effects of oxygen supply and Matrigel overlay. The reduction of oxygen consumption of hepatocytes in long-term culture is reported (Rotem et al., 1992), and it could gradually ameliorate ischemia in TCPS. Besides, in this experiment Matrigel was added in the medium and overlayed onto hepatocytes. It is known that the overlay of extracellular matrix (ECM), such as collagen and Matrigel, improves the condition of primary hepatocytes and makes bile canaliculi apparent and visible (Dunn et al., 1989; Moghe et al., 1996; Reif et al., 2015; Deharde et al., 2016; Gijbels et al., 2019). Interestingly, we also observed an extensive development of bile canaliculi and excessive bile secretion within primary hepatocytes when overlayed with Matrigel and cultured under sufficient oxygen supply, resulting in cholestasis-like hepatotoxicity in *in vitro* (data submitted elsewhere). This cholestasis-like hepatotoxicity under sufficient oxygen supply with Matrigel overlay may explain the slight decrease of albumin secretion observed in Figure 4. In turn, however, this suggests the possibility that so-called sandwich-culture of hepatocytes under sufficient oxygen supply may improve the amount of bile recovery (Marion et al., 2012). Overall, these results indicate the compatibility of PMP with primary hepatocyte cultures.

Finally, we performed a CYP activity test by using the substrate cocktail solution as reported previously (Kozakai et al., 2014; Scheidecker et al., 2020; Shinohara et al., 2021). Under cell-free condition, we found that substantial sorption into PDMS quickly proceeded just within 4 h of exposure, regarding most of the tested substrates (Figure 6). This result clearly demonstrates that an accurate evaluation of hepatic metabolisms in PDMS is almost impossible due to the immediate sorption of chemicals. In contrast, in PMP and TCPS wells, most of the tested substrates except for Bupropion and Amodiaquine were retained in the solution for 48 h of exposure. Interestingly, although Bupropion and Amodiaquine were largely sorbed even to both PMP and TCPS after 24 h, sorption at 4 h on both materials was almost negligible, suggesting that it is not critical for CYP activity test. Overall, sorption to PMP was shown to be comparable with TCPS, and much less than that to PDMS. Under the cell culture condition, among the substrates which showed a substantial sorption to PDMS, such as Bupropion, Amodiaquine, Bufuralol, and Midazolam, their metabolites were detected much less in the PDMS culture than in others, as expected (Figures 8C,D,G,H). Importantly, even as for substrates that showed a comparable sorption to all materials, such as Phenacetine and Diclofenac, the concentrations of metabolites were less in PDMS and TCPS, and prominently high in the PMP culture (Figures 8A,E), suggesting major sorption of metabolites into PDMS, and enhanced metabolic activity under sufficient oxygen supply in PMP.

As the results described previously clearly demonstrated, high oxygen permeability and low sorption characteristics of PMP successfully improved the quality of cultured primary hepatocytes and the accuracy of evaluation of hepatocyte metabolisms. While oxygen permeabilities are very low (Merkel et al., 2000; Uenishi et al., 2014), other thermoplastic elastomers and thermoplastics with less sorption characteristics than PDMS have been reported (Gencturk et al., 2017; Schneider et al., 2021). They are also promising alternatives to PDMS, and thus, an appropriate selection of materials seems important depending on the context of use (Campbell et al., 2021). Compared with PDMS, PMP is also transparent and exhibits the least autofluorescence, making it compatible with immunofluorescence observations as represented in the results. However, one of the drawbacks of PMP is its current availability. Until it is available on the market in the near future, only samples in the multi-well plate format are available upon request. Another drawback is compatibility with in-house fabrication methods. PMP may have high formability due to its hardness, but is not compatible with commonly used fabrication methods, such as soft lithography. This may be problematic especially when the focus is on constructing microfluidic devices. One solution may be hybrids with other materials, such as glass and polycarbonate. Microchannels can be fabricated with other materials and PMP sheets can be attached to the other side to complete the microchannels. Despite of some drawbacks as mentioned previously, in the scope of constructing a simple *in vitro* drug test system by using primary hepatocytes, we demonstrated that simple multi-well plates with a PMP bottom were superior to either PDMS- and TCPS-bottom plates. We thus conclude that PMP is a very promising material for accurate and reliable drug testing systems and a superior alternative to both PDMS and TCPS.

## Data Availability

The raw data supporting the conclusions of this article will be made available by the authors, without undue reservation.
