# Supplementary material for: Accurate Evaluation of Hepatocyte Metabolisms on a Noble Oxygen-Permeable Material With Low Sorption Characteristics
Source: Front Toxicol. 2022 Jun 6;4:810478. doi: 10.3389/ftox.2022.810478 (PMC9208656; doi:10.3389/ftox.2022.810478)
Supplement: Supplementary file 1 [file DataSheet1.pdf]

Figure S1.

Supplementary Figures

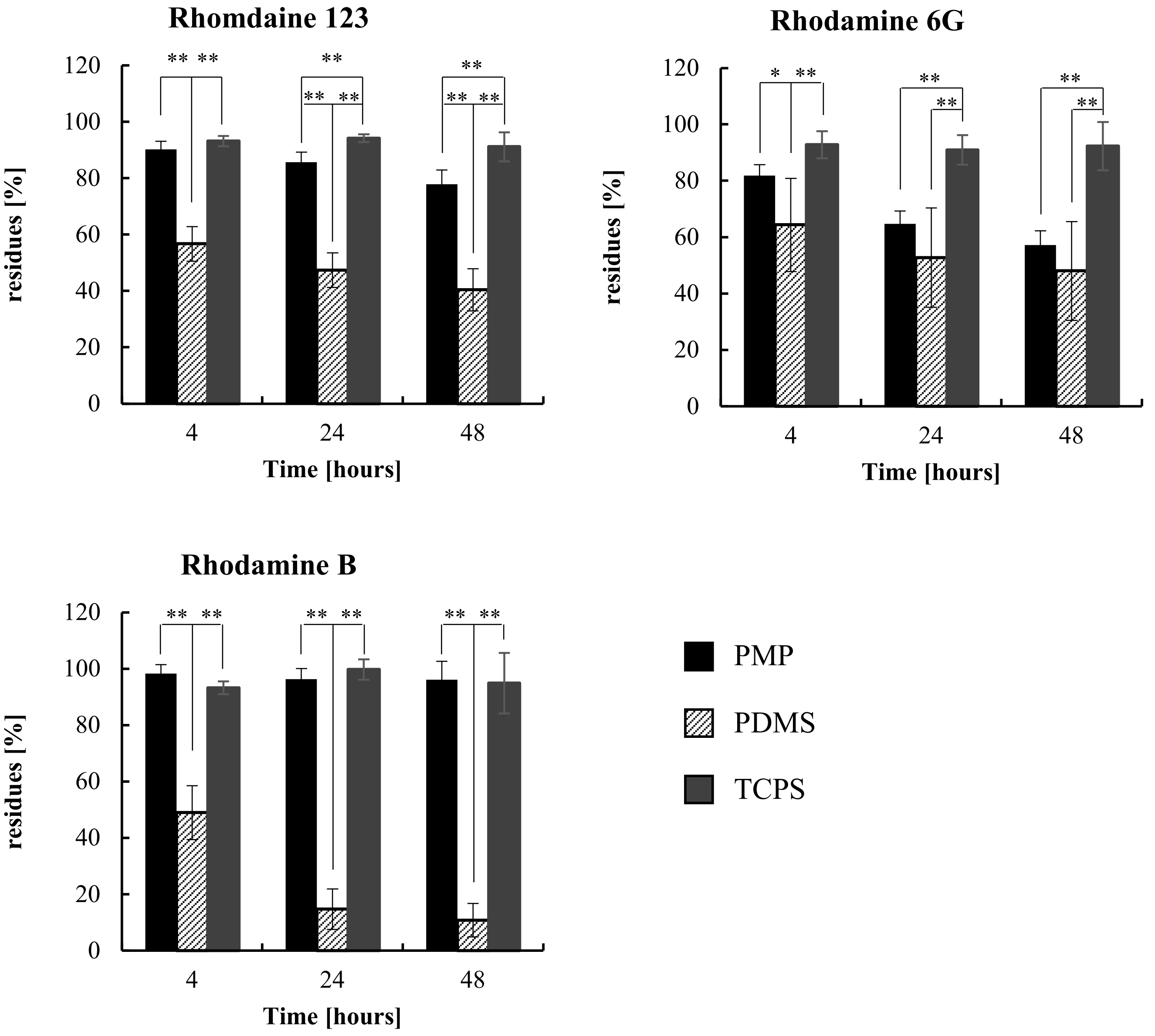

Figure S1. Statistical analysis of the data presented in Figure 2: the transition of the concentration of (a) Rhodamine 123, (b) Rhodamine 6G, (c) Rhodamine B, respectively. (N =6)

Table S1.

Table S1. Composition of hepatocyte culture medium

Component	Final concentration	Catalog number (Manufacturer)
Williams' E Medium	―	A1217601 (Gibco, USA)
Sodium Pyruvate (100X)	1 mM	11360070 (Gibco, USA)
GlutaMAX (100X)	2 mM	35050061 (Gibco, USA)
HEPES pH 7.4 (1 M)	15 mM	H3375 (Sigma, USA)
Penicillin-Streptomycin	0.5X	10378016 (Gibco, USA)
ITS+ (100X)	1X	354352 (Corning, USA)
Nicotinamide (5 M)	0.2 mM	N0636 (Sigma, USA)
Dexamethasone (10 mM)	0.1 μM	041-18861 (Wako, Japan)
mEGF	20 ng/mL	SRP3196 (Sigma, USA)
Ascorbic Acid (0.5 M)	0.5 mM	013-12061 (Wako, Japan)

Table S2.

Table S2. qRT-PCR primer sequences

No.	Gene target	Sequence (Forward, Reverse)	Size (bp)
1	β Actin (*β act*)	5'-ACAACCTTCTTGCAGCTCCT-3'<br>5'-CCCATACCCACCATCACACC-3'	196
2	Cytochrome P450 1A1 (*Cyp1a1*)	5'-AGTTCAGTCCTTCCTCACAGC-3'<br>5'-AATGTGGTGACGGCCAAGAG-3'	108
3	Cytochrome P450 1A2 (*Cyp1a2*)	5'-GTTCAAGCACAGTGAGAACTACA-3'<br>5'-ATCTCTGCCAATCACCGTGT-3'	199
4	Cytochrome P450 3A2 (*Cyp3a2*)	5'-GGGCCCTGCCGAGTAAG-3'<br>5'-AATCGTCACTACTGACCCTTTG-3'	179
5	Uridine diphosphate glucuronosyltransferase 1A1 (*Ugt1a1*)	5'-AGGACCTTCTGAGTCCTGCATC-3'<br>5'-CCGGAGGCGTTGACATAGG-3'	160
6	Bile salt export pump (*Bsep*)	5'-GGCCATTGTGCGAGATCCTA-3'<br>5'-CCACTCCTTGTGACACGACA-3'	188
7	Multidrug Resistance Transporter 2 (*Mrp2*)	5'-TCGAGAGAGGCTGACCATCA-3'<br>5'-CAAGCTGTAGGCCAGACACA-3'	115
8	Multidrug Resistance Transporter 3 (*Mrp3*)	5'-TGACCTGGAGACTGATGACCT-3'<br>5'-AAGATGCCTCCAGCTGCAAT-3'	183

Figure S2.

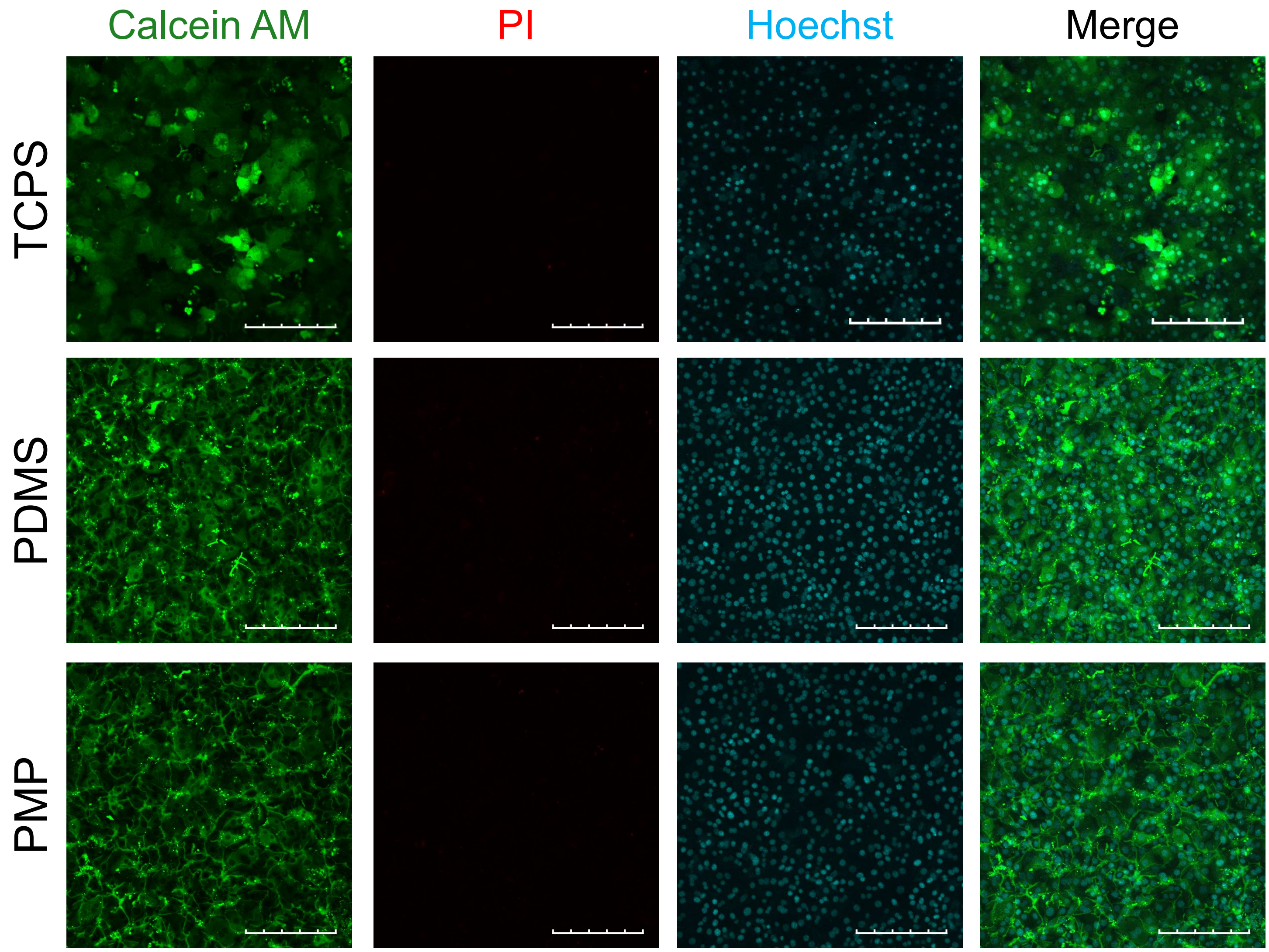

Figure S2. Cells were stained on day 3 using 1 μM Calcein AM, 4.5 μM ethidium homodimer-1, and 1 μg/ ml Hoechst 33342 (Dojindo Laboratories) in William's E culture medium (Gibco) without FBS. Live cells, dead cells, and nuclei were observed by confocal microscopy (Olympus, Tokyo, Japan, 20x objectives, scale bar = 200 μm) and indicated with green, red, and blue, respectively.

Figure S3.

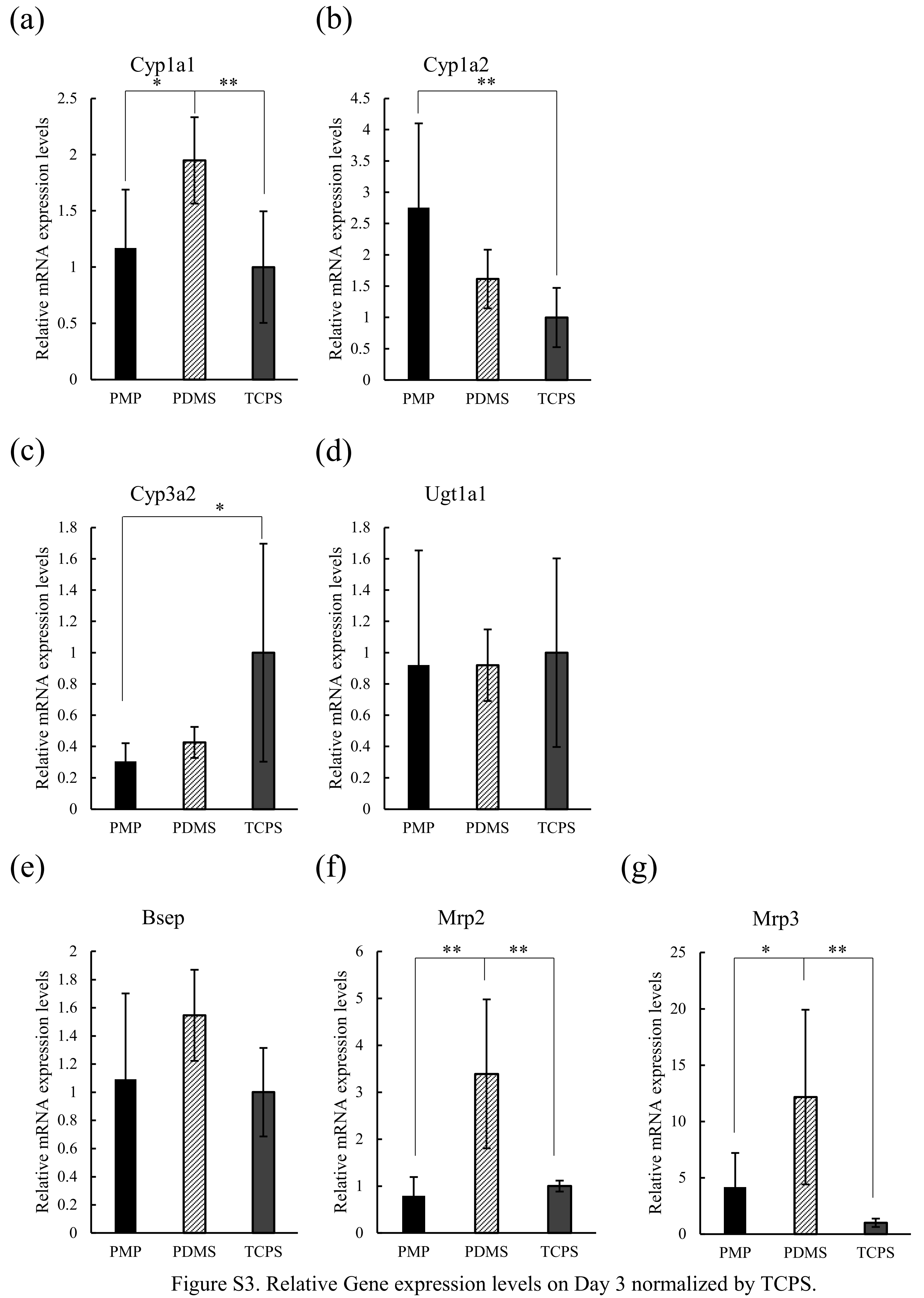

Figure S3. Relative Gene expression levels on Day 3 normalized by TCPS.
(*: $p<0.1$, **: $p < 0.01$, $N = 6$)

Figure S4.

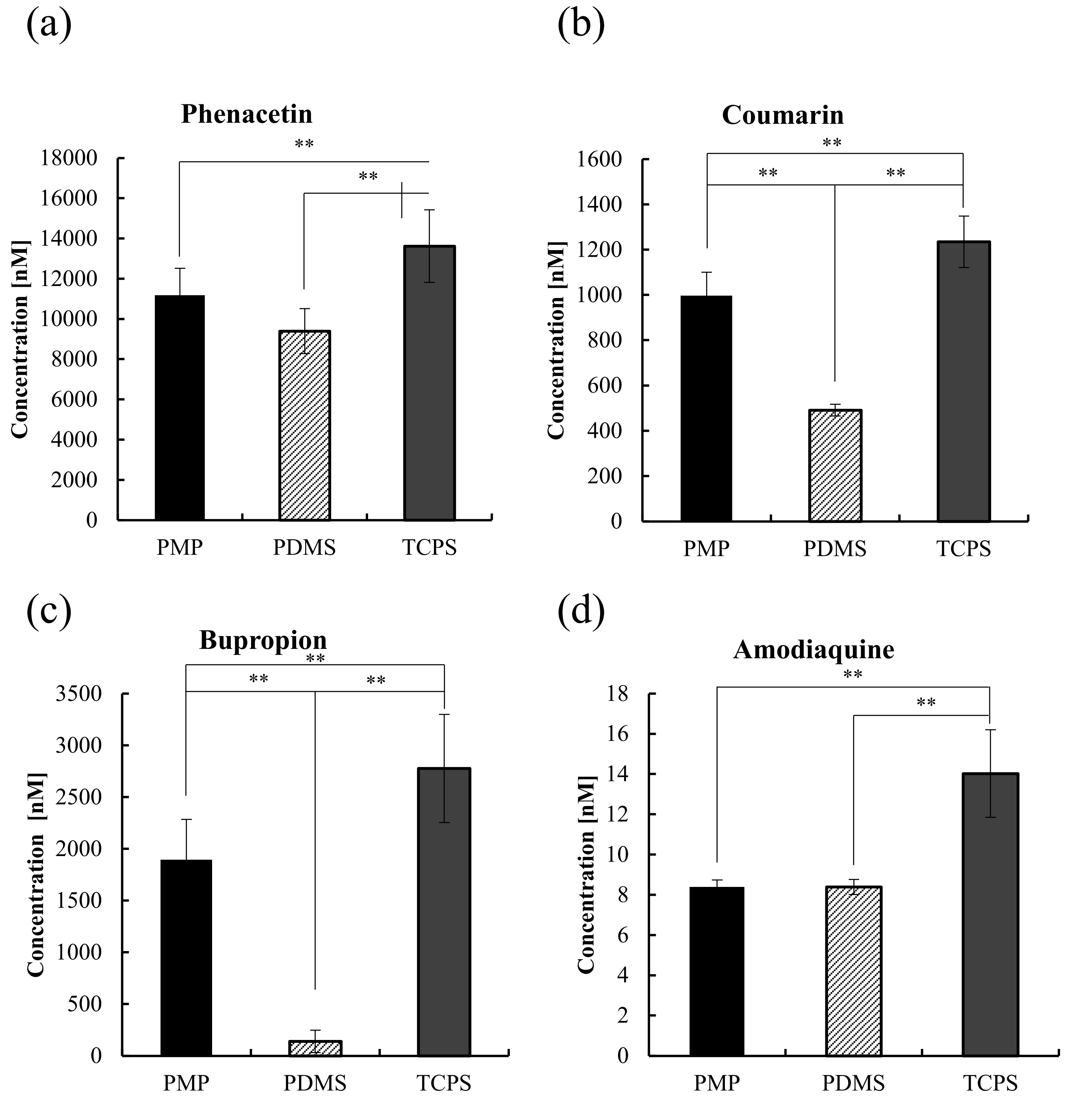

(a)
Phenacetin
Concentration [nM]
0
2000
4000
6000
8000
10000
12000
14000
16000
18000
PMP
PDMS
TCPS
**
**
(b)
Coumarin
Concentration [nM]
0
200
400
600
800
1000
1200
1400
1600
PMP
PDMS
TCPS
**
**
**
(c)
Bupropion
Concentration [nM]
0
500
1000
1500
2000
2500
3000
3500
PMP
PDMS
TCPS
**
**
**
(d)
Amodiaquine
Concentration [nM]
0
2
4
6
8
10
12
14
16
18
PMP
PDMS
TCPS
**
**

Figure S4.

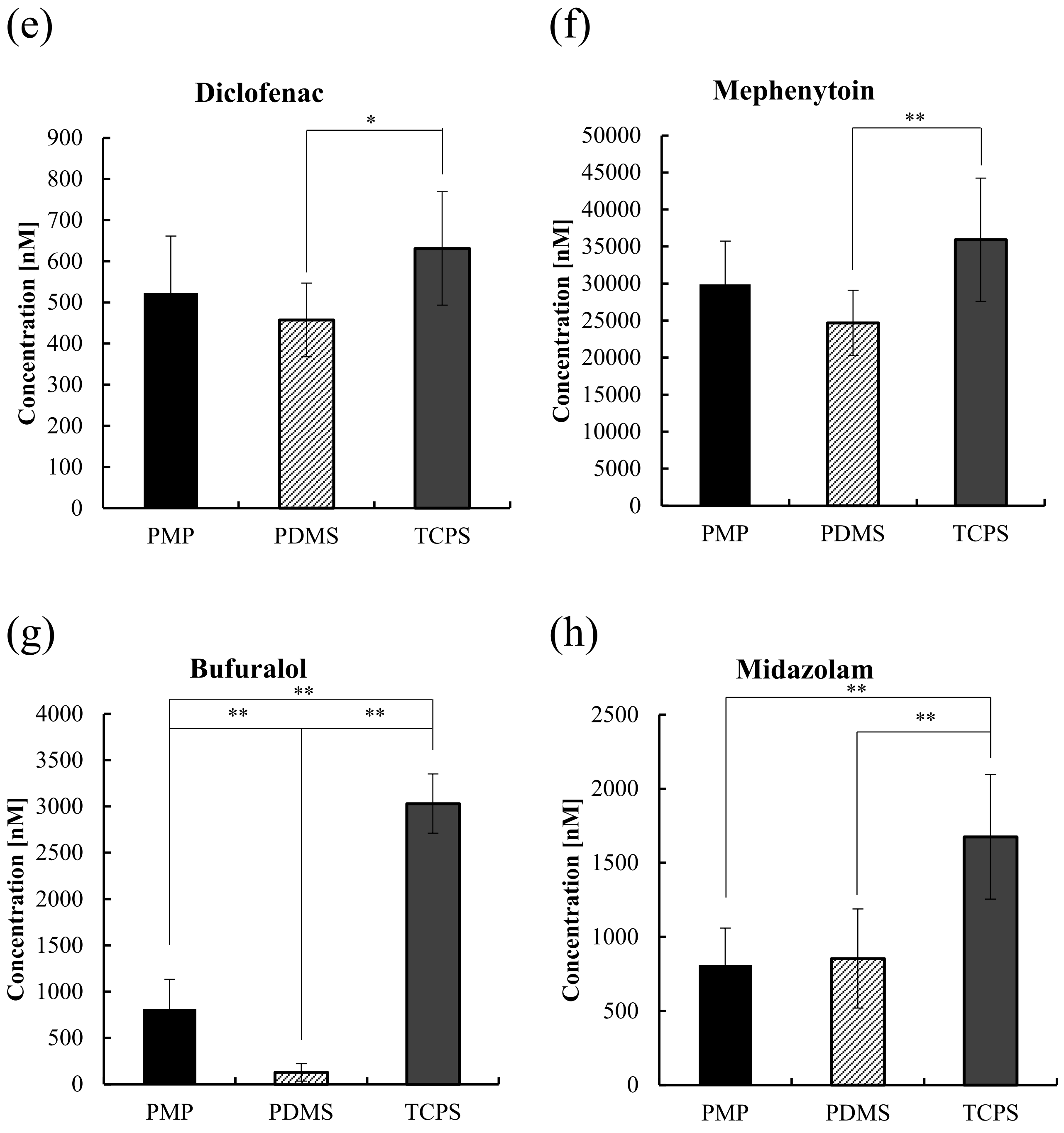

Figure S4. The concentrations of CYP substrates at 4h of exposure to cultured hepatocytes on Day 3. (a) CYP1A2, (b) CYP2A6, (c) CYP2B6, (d) CYP2C8, (e) CYP2C9, (f) CYP2C19, (g) CYP2D6, (h) CYP3A2 (N = 8)

Figure S5.

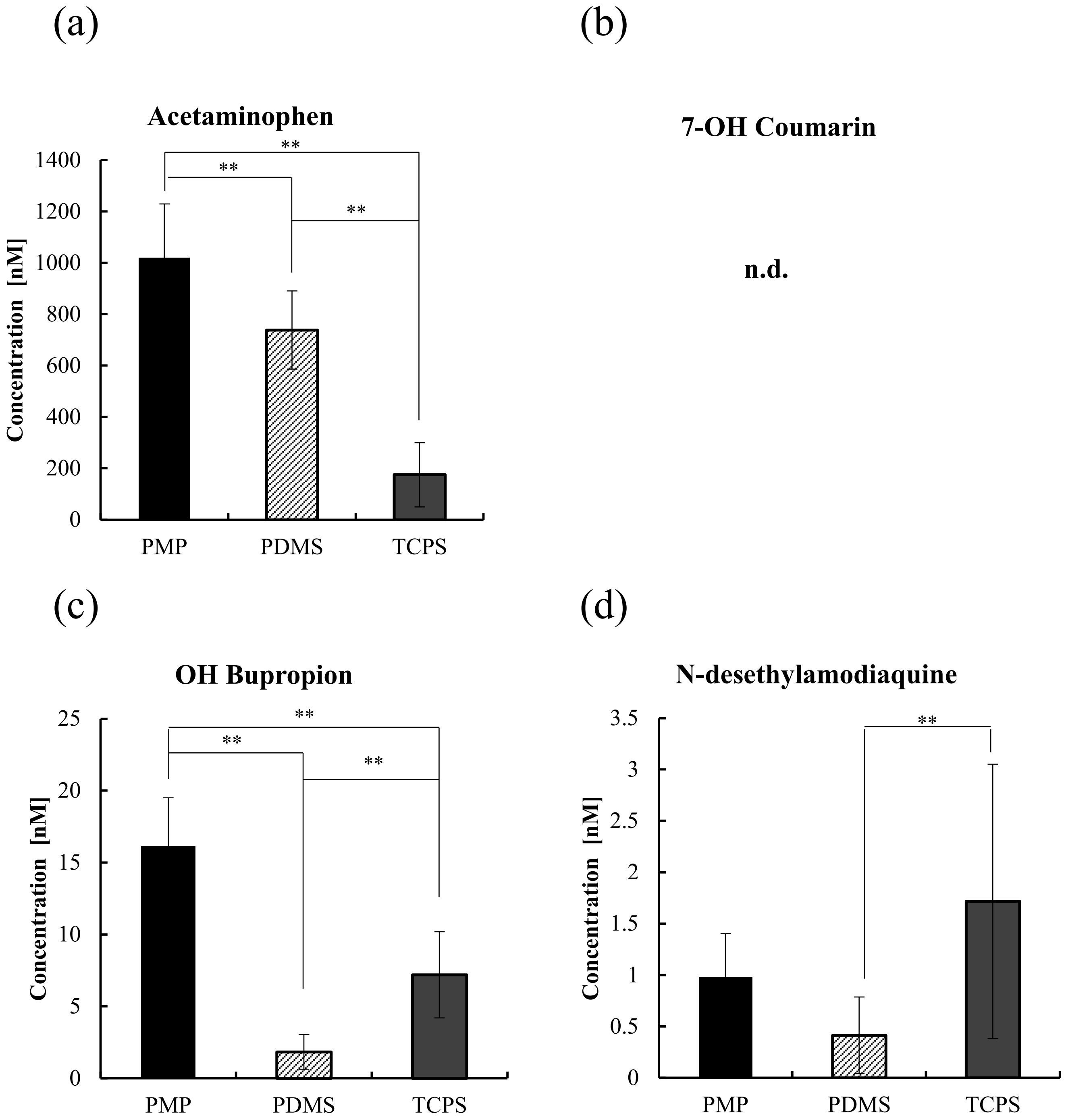

(a)
Acetaminophen
Concentration [nM]
1400
1200
1000
800
600
400
200
0
**
**
**
PMP
PDMS
TCPS
(b)
7-OH Coumarin
n.d.
(c)
OH Bupropion
Concentration [nM]
25
20
15
10
5
0
**
**
**
PMP
PDMS
TCPS
(d)
N-desethylamodiaquine
Concentration [nM]
3.5
3
2.5
2
1.5
1
0.5
0
**
PMP
PDMS
TCPS

Figure S5.

(e)

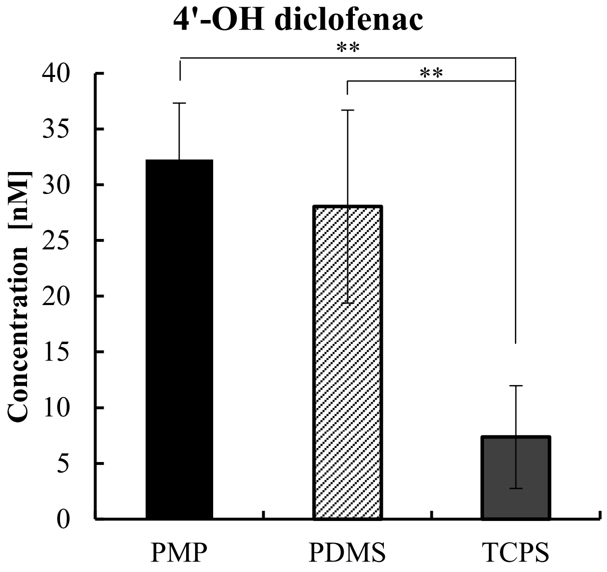

(f)

4'-OH Mephenytoin

n.d.

(g)

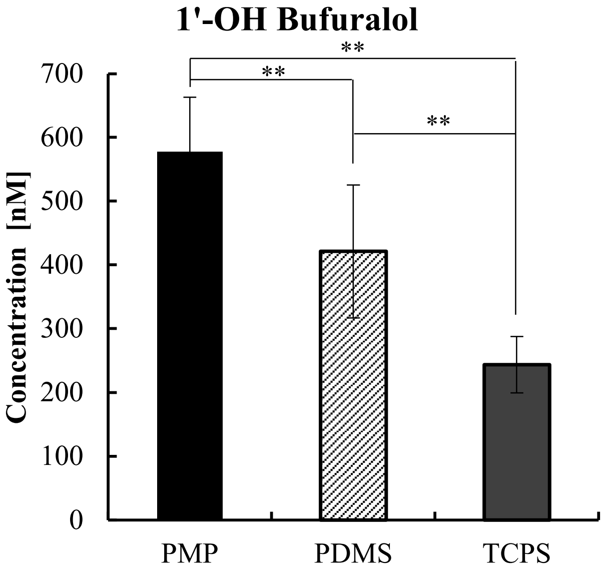

(h)

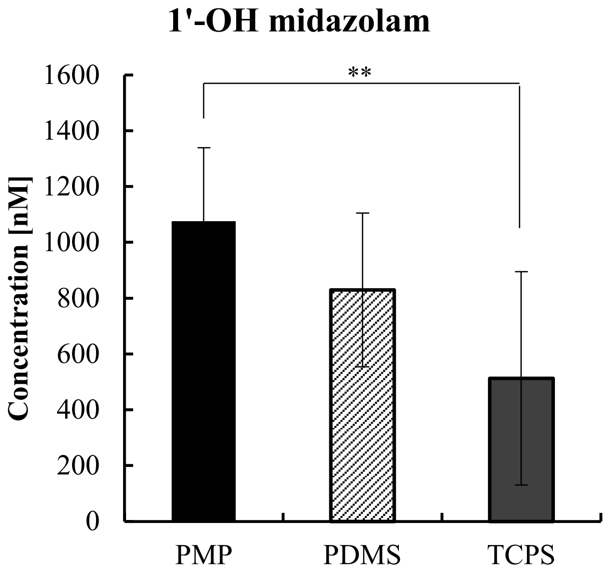

Figure S5. The concentrations of CYP metabolites at 4h of substrate exposure to cultured hepatocytes on Day 3. (a) CYP1A2, (b) CYP2A6, (c) CYP2B6, (d) CYP2C8, (e) CYP2C9, (f) CYP2C19, (g) CYP2D6, (h) CYP3A2 (N = 8)

Figure S6.

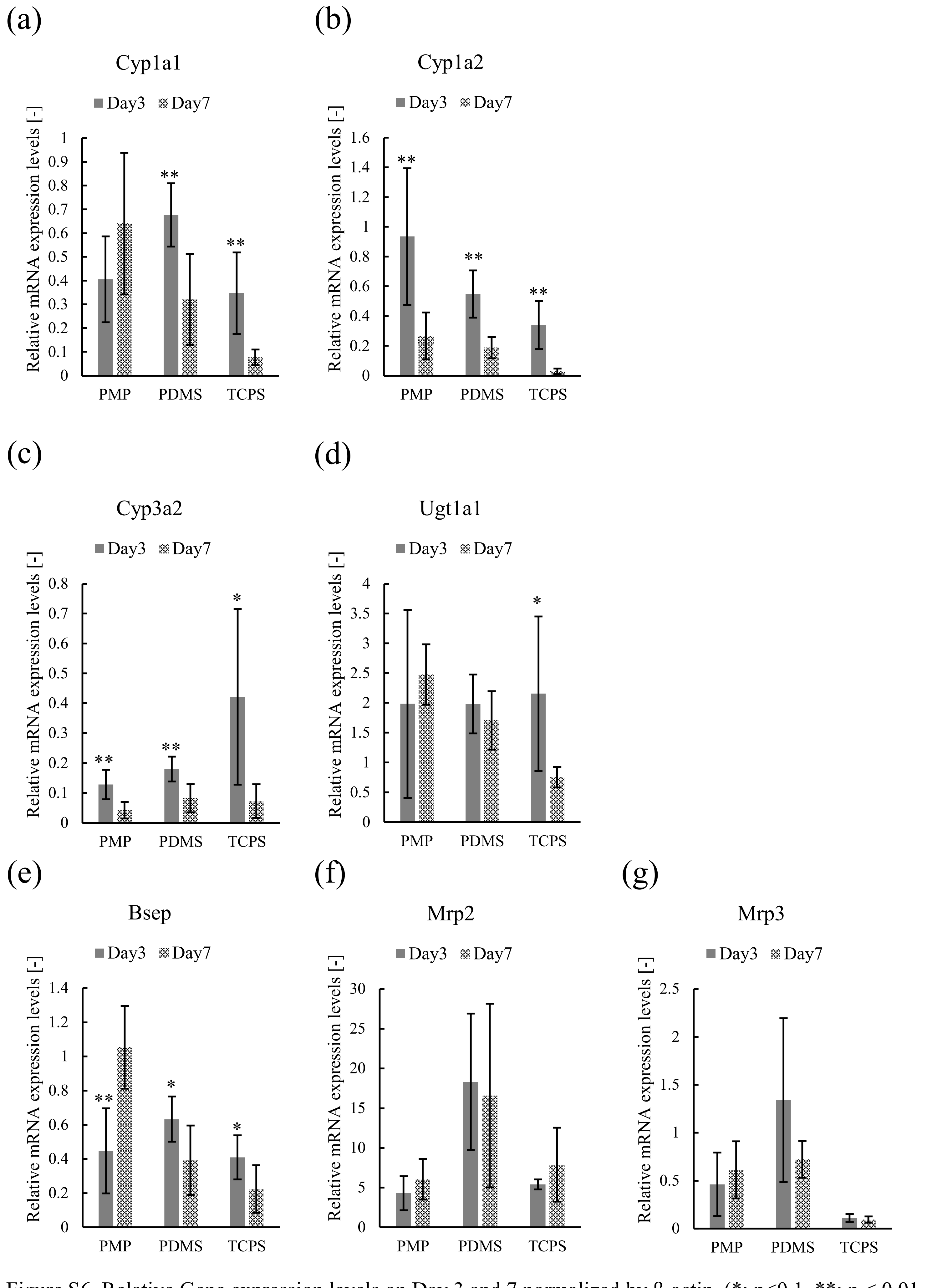

Figure S6. Relative Gene expression levels on Day 3 and 7 normalized by β-actin. (*: $p<0.1$, **: $p < 0.01$, $N = 6$, pairwise comparisons between Day 3 and 7 were conducted via Student's t-Test)
